# Micro-Rheological Changes of Red Blood Cells in the Presence of an Arterio-Venous Fistula or a Loop-Shaped Venous Graft in the Rat

**DOI:** 10.3389/fphys.2020.616528

**Published:** 2020-12-18

**Authors:** Balazs Szabo, Bence Tanczos, Adam Varga, Barbara Barath, Souleiman Ghanem, Zsofia Rezsabek, Mohammad Walid Al-Smadi, Norbert Nemeth

**Affiliations:** ^1^Department of Operative Techniques and Surgical Research, Faculty of Medicine, University of Debrecen, Debrecen, Hungary; ^2^Doctoral School of Clinical Medicine, University of Debrecen, Debrecen, Hungary

**Keywords:** hemorheology, microcirculation, red blood cell damage, arterio-venous fistula, microsurgery, anastomoses, micro-rheology

## Abstract

**Introduction:** In case of kidney failure, hemodialysis is the primary kidney replacement technique. Several vascular access methods used for the therapy, one of which is the arterio-venous fistula (AVF). In the AVF, the blood flow is altered, which can elevate the mechanical stress on the red blood cells (RBCs). This can affect the RBC hemorheological properties, and it can further cause systemic changes. To lower the turbulence and shear stress, we performed a loop-shaped arterio-arterial venous interposition graft (loop-shaped graft) to compare its effect to the conventional AVF.

**Materials and Methods:** Thirty male Wistar were used (permission registration Nr.: 25/2016/UDCAW). The animals were randomly divided into sham-operated, AVF, and loop groups (*n* = 10/each). The superficial inferior epigastric vein (SIEV) was used to create the AVF and the loop-shaped graft. Blood samples were taken before/after the surgery and at the 1st, 3rd, and 5th postoperative weeks. We measured hemorhelogical, hematological, and blood gas parameters. The microcirculation of the hind limbs was also monitored using Laser Doppler fluxmetry.

**Results:** Hematocrit, RBC count, and hemoglobin decreased by the 1st postoperative week. The erythrocyte aggregation values significantly increased in the fistula group by the 5th week (6.43 ± 2.31 vs. 13.60; *p* < 0.0001; vs. before operation). At the postoperative 1st week in the loop group, the values showed a significant decrease in RBC deformability. During the maturation period, dominantly at the 5th week, all values were normalized. The operated hind limb’s skin microcirculation significantly increased in the sham and loop group by the 1st week (39 ± 10.57 vs. 73.93 ± 1.97 BFU, *p* < 0.01). This increase wasn’t observed in the fistula group probably due to a steal-effect.

**Conclusion:** Unlike in the loop group, in the presence of the fistula, several rheological parameters have changed. The loop-shaped graft had only minimal impact on micro-rheological parameters.

## Introduction

In the microcirculation, the red blood cells’ (RBCs) hemorheological parameters can affect the perfusion of the supplied tissue (Chien, 1987; Jung et al., 2011; Jose et al., 2014; Muravyov et al., 2018). These parameters can be affected by several factors, for example, the body temperature, the pH of the blood, medication, age, inflammation, etc. (Aarts et al., 1986; Hughes and Kikuchi, 1988; Lecklin et al., 1996; Kuzman et al., 2000; Yao et al., 2003; Yang et al., 2014; Bateman et al., 2017; Nemeth et al., 2018; Somogyi et al., 2018). The RBC deformability and aggregation during infection can be altered due to the pathogen itself like in the case of malaria or due to the inflammation (Hosseini and Feng, 2012; Nemeth et al., 2015). In sepsis, the early signs of the inflammation could be shown by the hemorheological changes (Nemeth et al., 2015). Metabolic diseases like diabetes also have a great effect on the deformability of the RBCs, showing a great difference between patients with diabetes who are therapeutically well-balanced compared to patient who are not (Kiesewetter et al., 1986).

Mechanical trauma can also be damaging the blood cells and, therefore, it can affect the micro-rheological properties of the RBCs. This can be caused by an external source like running, and in extreme conditions, it can even cause hemolysis (Telford et al., 2003). The internal source of mechanical trauma is caused by altered flow due to vascular surgery or some pathological processes, such as stenosis. In case of stenosis, the laminar flow changes to semi-turbulent or turbulent flow distal to the narrowing (Clark, 1980; Liu, 2007). After surgery, the anastomosis rarely has the exact same geometry of the original vessel, and during healing, the geometry of the vessel can further change. Implanted objects like heart valve and graft can also disturb the blood flow (Karanasiou et al., 2015; Vahidkhah et al., 2016). The heart valve can even cause hemolysis but not to the extent of anemia. The location and the number of heart valves greatly affect the amount of hemolysis that occurs (Skoularigis et al., 1993). The main source of mechanical trauma in the previously mentioned cases is the appearing turbulence, which consequently elevates the shear-stress as well (Ku, 1997). The turbulent flow and elevated shear stress are damaging to the RBCs depending on the magnitude and the exposure time (Kameneva et al., 2007; Yen et al., 2014).

High flow velocity together with turbulent flow and high shear stress should be avoided because of the previously mentioned reasons. However, in case of an arterio-venous fistula (AVF), these are the given conditions especially after maturation (Anderson et al., 1977; Fitts et al., 2014; Pike et al., 2017; Oliver, 2018). At the beginning of the formation of the fistula, the flow drastically changes compared to the physiological venous condition, the blood flow is high, and so the shear stress. The vein reacts to the new environment and the diameter increases to the more favorable conditions, which are lower shear stress and blood velocity. The venous endothelial tissue has a key role mediating the previously mentioned histological changes (Browne et al., 2015). If the vein graft is inserted into an artery, the conditions will be different. In this case, the vein graft experiences almost physiological arterial flow and shear stress. In these conditions, the vessel wall will adapt differently and the diameter will not change drastically, and it can be increased or decreased depending on the blood flow velocity and shear stress profile (Zarins et al., 1987; Binns et al., 1989; Gnasso et al., 1996). The flow profile of the venous and arterial blood flow regarding mean flow velocity and flow volume in equal-sized vessels are relatively similar, and the key difference is the much higher pressure in the arteries (Ku, 1997; Holland et al., 1998; Libertiny and Hands, 1999; Thiriet, 2015).

The mean blood flow velocity, the peak velocity, and the flow volume can drastically change after AVF surgery, both in the arterial and venous side (Anderson et al., 1977). Also, in the fistula itself, because of the high pressure gradient, the vein experiences extreme conditions including elevated turbulent flow and high shear stress (Hammes, 2015). After the fistula is made, it constantly affects the individual RBCs until it is thrombotized or surgically reconstructed. The effect of the disturbed flow on the RBCs has not been completely studied yet in case of AVF. Also, the systemic effect of the circulating damaged cells is not known. It is important because when the microcirculation is impaired due to a disease like diabetes or small vessel vasculitis, a creation of an AVF can further decrease the already damaged distal microcirculation (Savage et al., 2000; Rask-Madsen and King, 2013). Damaged RBCs circulating in the vascular system could significantly decrease the microcirculation of the distant tissues. The performed fistula can also cause permanent perfusion problem of the operated limb due to steal mechanism (Chai et al., 2018). The turbulent flow and the shear stress can be modified with altering the shape of the vessel or the graft (Karino et al., 1987; Coppola and Caro, 2009).

We hypothesized that the gradual curvature of a loop-shaped graft instead of the traditional U-shape could be beneficial to an AVF. Also to reduce the flow velocity and the shear stress in the vessel, we decided to have the vessel implanted into the artery thus making a loop-shaped arterio-arterial venous interposition graft (loop-shaped graft). That is why we aimed to investigate how an AVF affects the local and systemic microcirculation and hemorheological parameters compared to a loop-shaped graft.

## Materials and Methods

### Experimental Animals

Thirty male Wistar (Crl:WI) rats were used (ethical permission registration Nr.: 25/2016/UDCAW). The average age of the animals was 8–10 weeks and 349.7 ± 13.76 g were the average weight. They were randomly divided in to three groups, as sham-operated (*n* = 10), fistula (*n* = 10), and loop group (*n* = 10). The rats were kept in standard cages and fed with *ad libitum* with commercially available food and water.

### Surgical Protocol

All the rats were anesthetized using a mixture of ketamine (100 mg/kg), xylazin (10 mg/kg), and atropine (0.05 mg/kg), administered intraperitoneally (Green et al., 1981). For thrombosis, prophylaxis heparin was administered i.v. (80 IU/kg). After the anesthesia, the lower abdominal wall and both inner thighs were shaved and disinfected using Betadine solution. A 26-gauge cannula was inserted into the lateral tail vein for blood sampling and fluid therapy. The surgical site was carefully isolated with gauze and an incision was made above the right inguinal ligament, and then the femoral vessels were isolated. The superficial inferior epigastric vein (SIEV) was also isolated, which is the second side branch of the femoral vein on the medial side from the inguinal ligament. The previously mentioned steps were the same in each group. A 10/0 non-absorbable polyamide thread was used with 3/8 serosa (taper) needle for every anastomosis, and a 6/0 absorbable polyglycolic acid thread was used for the skin sutures with 3/8 cutting needle. In case of the sham-operated group, after the dissection, the rat stayed under anesthesia for an additional 90 min, which was the duration of the vascular intervention in the other groups. Then the skin was closed with a continuous suture.

**Figure 1:**
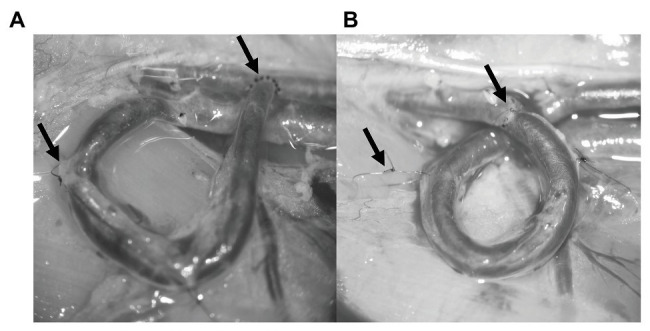
Photos about the finished vascular interventions. The left arrows on each panel show the anchoring stitches, and the right arrows show the anastomosis. **(A)** U-shaped arterio-venous fistula. **(B)** Loop-shaped arterio-arterial venous interposition graft.

The fistula was performed after the dissection using the SIEV. The vein was mobilized, but a significant amount of connective tissue was left intentionally on the vessel wall. This was useful in a later part of the surgery. The distal end of the SIEV was ligated, cut, and finally flushed with heparin solution. After that the vessel was positioned by bending the SIEV 180° in the shape of U. A side cut was made on the femoral artery forming approximately the same diameter orifice as the SIEV, and it was also washed with heparin solution. The vein was sutured to the orifice on the femoral artery using simple interrupted stitches, so creating and end-to-side anastomosis (Figure 1A, right arrow). We checked the flow of the fistula by closing the SIEV with a microvascular clip. By releasing the clip, arterial blood entered the femoral vein and changed the color of the vessel bright red. Two anchoring sutures were used to secure the U-shape of the fistula. The stitches were placed into the connecting tissue around the vessel and into the gracilis muscle (Figure 1A, left arrow). The skin was sutured in the same fashion as in the sham-operated group.

In case of the loop instead of the side-to-end anastomosis, two end-to-end anastomoses were performed using simple interrupted stitches. First, the SIEV was mobilized and washed just like in the fistula group, and then the femoral artery was cut in half and each vessel end was flushed with heparin solution. The start of the loop making process was to connect the distal end of the SIEV to the proximal end of the artery. After the anastomosis was made, it was tested for leakage and flow. Since the proximal end of the SIEV was still connected to the femoral vein, at this point, it was acting as an AVF and, therefore, it was tested in that manner. After the completion of the previous steps, the proximal end of the vein was ligated and cut at the junction of the SIEV and the femoral vein. The distal end of the femoral artery and the proximal end of the SIEV was also sutured forming an end-to-end anastomosis (Figure 1B, right arrow). All the microvascular clips were removed, and the arterio-arterial graft was tested for patency and leakage. The patency was tested using the double occlusion test (“milking” test; Pandya et al., 2003). At this stage, the venous graft was twisted so that it would be shaped like a loop. This was fixed with two anchoring stitches just like in the case of the fistula (Figure 1B, left arrow). The skin was sutured as it was mentioned previously. During the postoperative weeks, flunixin was used for pain management (10 mg/kg, s.c.). At the 5th postoperative week under general anesthesia, the vessels were dissected again for visual inspection and for patency testing.

### Blood Sampling Protocol

Blood samples were taken from the tail cannula before/after the surgery and at the 1st, 3rd, and 5th postoperative week (PO1, PO3, and PO5 week). The blood was stored in vacutainer tubes (BD Vacutainer® tubes, 5.4 g K3-EDTA, 3 ml). Each time 300 μl of blood was taken for the hematological and hemorheological measurements, plus an additional 90 μl was taken before and after the surgery and also at the PO5 week for blood gas and blood electrolyte analysis. At the postoperative weeks, the rats were anesthetized with the same anesthetic mixture, and the lateral tail vein was cannulated again. At the PO5 week, blood was also taken from the inferior caval vein in the fistula group to test the composition of the mixed (arterio-venous) blood.

#### Hematological Variables

All the measurements were performed by a Sysmex K-4500 automate (TOA Medicor Electronics Co., Ltd., Japan). In this paper, we analyzed the RBC count (10^12^/L), the white blood cell (WBC) count (10^9^/L), the hematocrit [Hct (%)], the hemoglobin concentration [Hgb (g/L)], the mean corpuscular volume [MCV (fL)], and the platelet number [Plt (10^9^/L)].

#### Blood Gas, Acid-Base, Metabolic Parameters, and Electrolytes

We measured the partial oxygen and partial carbon dioxide pressure [*p*O_2_, *p*CO_2_ (mmHg)], the oxygen saturation [sO2 (%)], pH, bicarbonate [HCO_3_^−^ (mmol/l)], glucose [Glu (mmol/L)], and lactate [Lac (mmol/L)]. Also some blood electrolytes were measured: sodium, potassium, calcium, and chloride (mmol/L) using an EPOC® Blood Analysis System (Epocal Inc., Canada).

#### Red Blood Cell Deformability and Red Blood Cell Aggregation

The erythrocyte deformability was examined using the LoRRca MaxSis Osmoscan ektacytometer (Mechatronics BV, Netherlands). Ten microliter of blood was diluted in 2 ml of polyvinyl-pyrrolidone (PVP) and phosphate buffered saline (PBS) solution (viscosity: 27 mPas, osmolarity: 300 mOsm/kg, pH: ∼7.3). The deformability of the RBCs was tested by determining the elongation of the cell under increasing shear stress [SS (Pa)] based on laser-diffractometry. The elongation of the cells was recorded between 0.3 and 30 Pa. The range of SS could be set between 0.3 and 75 Pa, but the upper range is not physiological, which could show unrealistic values (Baskurt et al., 2009). A laser diffraction pattern was provided by shining a beam on the RBC suspension. The laser beam is scattered on the surface of the RBCs. The device analyses the pattern and the software calculates and presents the data as an elongation index (EI) – shear stress curves (SS; Hardeman et al., 2007). The values of the EI are proportional to the erythrocyte deformability. Using the Lineweaver-Burke analysis, the maximal EI (EI_max_) and the shear stress at half-maximal elongation [SS_1/2_ (Pa)], and their ratio (EI_max_/SS_1/2_) were also calculated. Erythrocyte aggregation was measured using a Myrenne MA-1 erythrocyte aggregometer (Myrenne GmbH, Germany), based on light-transmittance method (Hardeman et al., 2007). Approximately 20 μl of blood was required for each test. Aggregation indices as M 5 s and M 10 s were tested at 0 s^−1^ shear rate and M1 5 s and M1 10 s at 3 s^−1^ shear rate.

### Microcirculation

The microcirculation of the tissue was monitored using a Laser Doppler (LD) fluxmeter (LD-01, Experimetria Ltd., Hungary) and a standard pencil probe (Oxford Optronix Ltd., United Kingdom). The device expresses the blood flux unit (BFU), which is a dimensionless number as an integral over the number and velocity of RBCs in the given region (~1 mm^3^). This measurement was taken before, 10 min after ischemia and 10 min after reperfusion, and also at the 1st, 3rd, and 5th postoperative weeks. Six points were tested on each side of the animals: lower abdominal wall, the inner thigh, and the second metatarsal foot pad. For the data analysis S.P.E.L. Advanced Kymograph software (Experimetria Ltd., Hungary) was used. Ten seconds of LD recordings were recorded after the stabilization of the waves, averaging the values (Nemeth and Szabo, 2017). The surface temperature of the foot (°C) was also monitored using an infrared thermometer at the 5th postoperative week.

### Statistics

The statistical analyses were performed using the GraphPad Prism 8 software. The significance level was set to *p* ≤ 0.05. All data distribution was checked for normality, and accordingly, Student t-test or Wilcoxon or Mann-Whitney non-parametric tests, as well as two-way ANOVA tests were used. The correlation between data sets was examined by calculating the Pearson correlation coefficient.

## Results

### General Observations

After 5 weeks of maturation, all the loops arterialized and all the fistulas matured. Regardless of the surgical intervention, all operated hind limbs remained functional and had no visible difference compared to the other hind limbs.

### Hematological Variables

**Figure 2:**
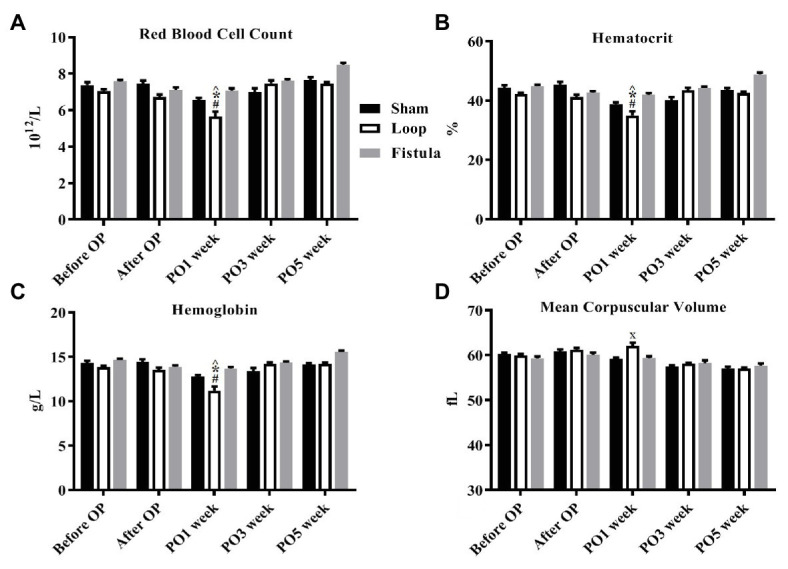
Changes of selected hematological values. **(A)** Red blood cell count; **(B)** hematocrit; **(C)** hemoglobin; and **(D)** mean corpuscular volume. *n* = 10, mean ± SEM, ^*p* < 0.05 vs. sham before operation, *p < 0.05 vs. loop before operation, #*p* < 0.05 vs. fistula before operation, *x* < *p*0.05 vs. loop and fistula on the PO1 week.

The hematological values showed the biggest changes at the PO1 week (Figures 2A–C). The Hct, RBC count, and the Hgb values decreased significantly within each group and the values normalized by the end of the PO5 week (Figures 2A–C). The loop group values were significantly lower at the PO1 week than the other groups (Figures 2A–C). MCV values also increased significantly at the PO1 week, this increase was notable compared to the other groups too (Figure 2D). Interestingly the Hct, RBC count, and Hgb showed a distinctive elevation at the PO5 week within the fistula group and also compared to the other two groups (Figures 2A–C). The Plt count significantly elevated in the loop group at the PO1 (Table 1). The WBC values increased after the surgery and significantly decreased in the sham and loop groups. In the fistula groups, the WBC count remained in higher level (Table 2).

**Table 1:** Platelet count (Plt) at PO1 and PO5 weeks.

Plt (10^9^/L)								
Group	PO1				PO5			
	Mean	±SD	*p*	Vs.	Mean	±SD	*p*	Vs.
Sham	841.08	98.76	**0.0278**	Loop	676.75	113.31	0.9995	Loop
Loop	950.43	236.26	0.1062	Fistula	675.22	85.46	0.8844	Fistula
Fistula	868.69	114.06	0.7788	Sham	697.36	67.23	0.882	Sham

The bold means that the p values are at significant level.

**Table 2:** Changes of white blood cell (WBC) count values.

WBC (10^9^/L)												
Group	Sham operated *n* = 10				Loop *n* = 10				Fistula *n* = 10			
	Mean	±SD	*p*	Vs.	Mean	±SD	*p*	Vs.	Mean	±SD	*p*	Vs.
Before	8.25	1.44	0.1543	PO1	7.83	2.20	0.8965	PO1	9.01	1.32	0.9857	PO1
After	4.91	1.68	**<0.0001**	PO1	7.57	1.87	0.6946	PO1	8.89	2.22	0.9516	PO1
PO1	9.62	0.96	0.0632	PO3	8.39	2.06	**0.0353**	PO3	9.31	2.30	0.2565	PO3
PO3	7.84	1.07	0.9955	PO5	6.34	1.58	0.9997	PO5	8.07	1.12	0.9983	PO5
PO5	7.58	0.62	**0.0216**	PO1	6.20	1.33	**0.026**	PO1	7.89	1.38	0.1388	PO1

The bold means that the p values are at significant level.

### Blood Gas, Acid-Base, Metabolic Parameters, and Electrolytes

**Figure 3:**
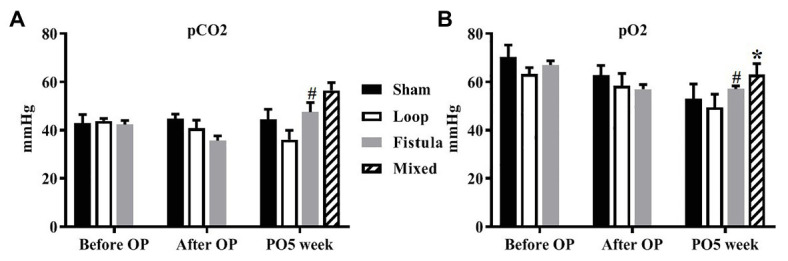
Changes of partial carbon dioxide pressure **(A)** and partial oxygen pressure **(B)**. *n* = 10, mean ± SEM, *p < 0.05 vs. fistula at the PO5 week, #*p* < 0.05 vs. fistula before operation.

Several parameters were measured but most of them did not show any notable changes during the observation period. Only the oxygen and carbon dioxide partial pressures changed significantly in the fistula group (Figures 3A,B). The *p*CO_2_ elevated after the surgery (after OP vs. PO5 week; 35.67 ± 5.81 vs. 47.54 ± 11.22; *p* = 0.0076), and accordingly the *p*O_2_ decreased (before OP vs. PO5 week; 67.08 ± 4.93 vs. 57.08 ± 3.19; *p* = 0.461). At the PO5 week, the fistula group had the highest *p*O_2_ values, but the change was not significant. We also took blood samples in the fistula group from the inferior caval vein, where the venous blood mixed with the arterial blood from the fistula. This was done at the PO5 week when we terminated the animals. Most notably the *p*O_2_ (venous vs. mixed; 57.083 ± 3.20 vs. 63.12 ± 11.14; *p* = 0.0099) and glucose concentration (venous vs. mixed; 18.95 ± 3.61 vs. 24.93 ± 2.31; *p* = 0.0109) were increased. Other acid-base or metabolic parameters and electrolytes did not change significantly.

### Red Blood Cell Deformability and Red Blood Cell Aggregation

**Figure 4:**
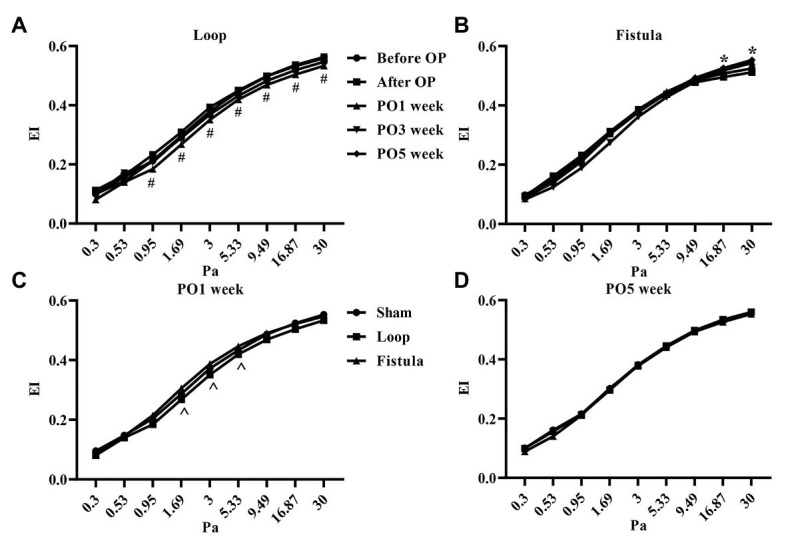
Changes of red blood cell deformability (elongation index values in the function of shear stress) in the loop group **(A)**, fistula group **(B)**, all groups at the PO1 week **(C)**, and all groups at the PO5 week **(D)**. *n* = 10, mean ± SEM, #*p* < 0.05 vs. PO3 week and PO5 week, *p < 0.05 vs. before operation, ^*p* < 0.05 vs. sham and loop group.

**Figure 5:**
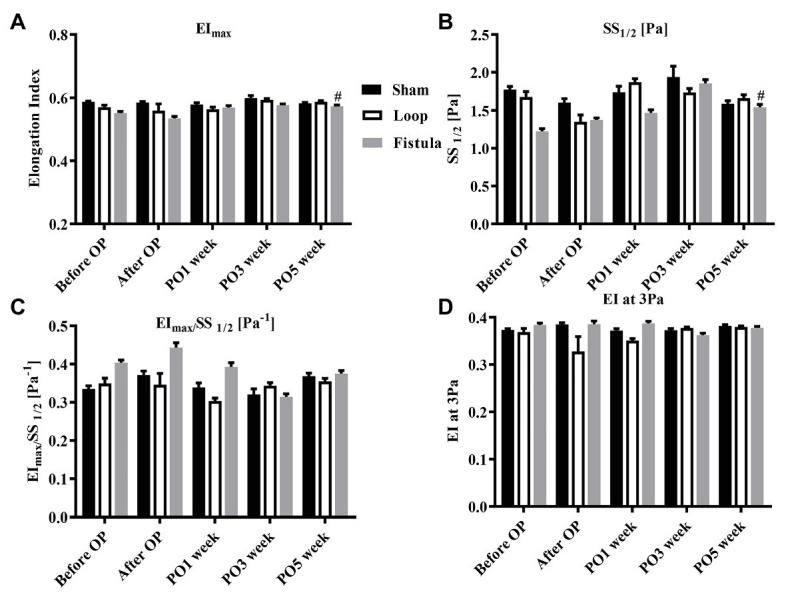
Alterations of maximal elongation index (EI_max_) **(A)**, shear stress at half-maximal elongation (SS_1/2_) **(B)**, their ratio **(C)**, and the elongation index at 3 Pa **(D)**. *n* = 10, mean ± SEM, #*p* < 0.05 vs. fistula before operation.

**Figure 6:**
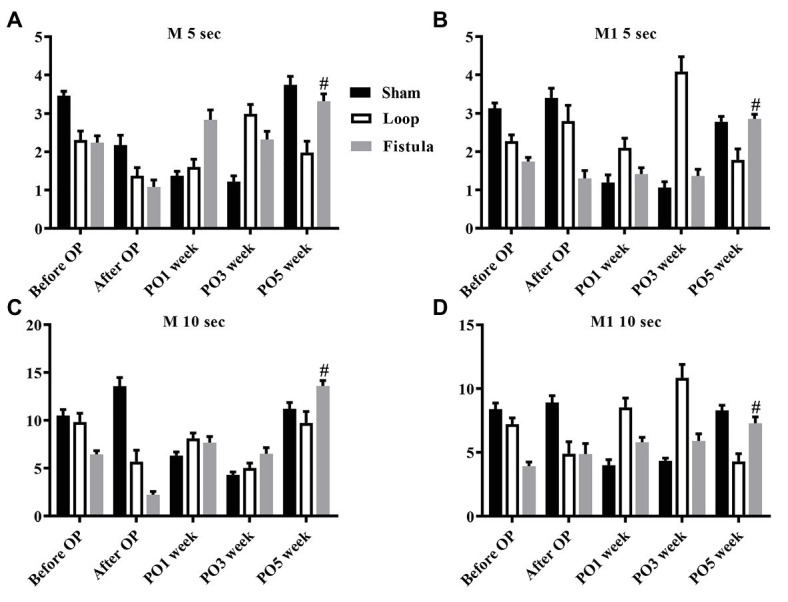
Changes in red blood cell aggregation indices: M 5 s **(A)**, M1 5 s **(B)**, M 10 s **(C)**, and M1 10 s **(D)**. *n* = 10, mean ± SEM, #*p* < 0.05 vs. fistula before operation.

In conjunction with the hematological result, the most prominent changes were observed at the PO1 week in the loop group (Figure 4A). The RBC deformability significantly impaired by the PO1 week in the loop group and compared to the other groups, the changes were significant (Figure 4C). The largest differences were seen between 1.69 and 5.33 Pa shear stress. However, in all groups, the deformability values were almost the same by the end of the postoperative period (Figure 4D). The fistula showed significant elevation at the PO5 week only above 16.87 Pa compared to the values before the operation (Figure 4B). The EI_max_ and EI_max_ /SS_1/2_ values also normalized at the end, except in the fistula group, where the values significantly increased (EI_max_: before OP vs. PO5 week; 0.55 ± 0.023 vs. 0.57 ± 0.015; *p* = 0.0015; Figures 5A,B). The other calculated values did not show notable changes (Figures 5C,D). In case of the RBC aggregation, the results showed a significant decrease after the surgery in the loop and fistula groups (Figures 6A–D). The sham-operated group values behaved irregularly after the surgery and returned back to the original range in sham-operated and loop groups, except in the fistula group, where the values were significantly higher at the PO5 week (Figures 6A–D). This was consistent with the hematological findings, because at the PO5 week, in the fistula group, the Hgb, RBC, and Htc values also significantly increased. Our other finding was that the aggregation values in the fistula group strongly correlated with the deformability values at higher shear stresses, namely at 9.49, 16.87, and 30 Pa (*p* = 0.0333, *R*^2^ = 0.8235; *p* = 0.0095, *R*^2^ = 0.9218; *p* = 0.0207, *R*^2^ = 0.8712, respectively).

### Microcirculation

**Figure 7:**
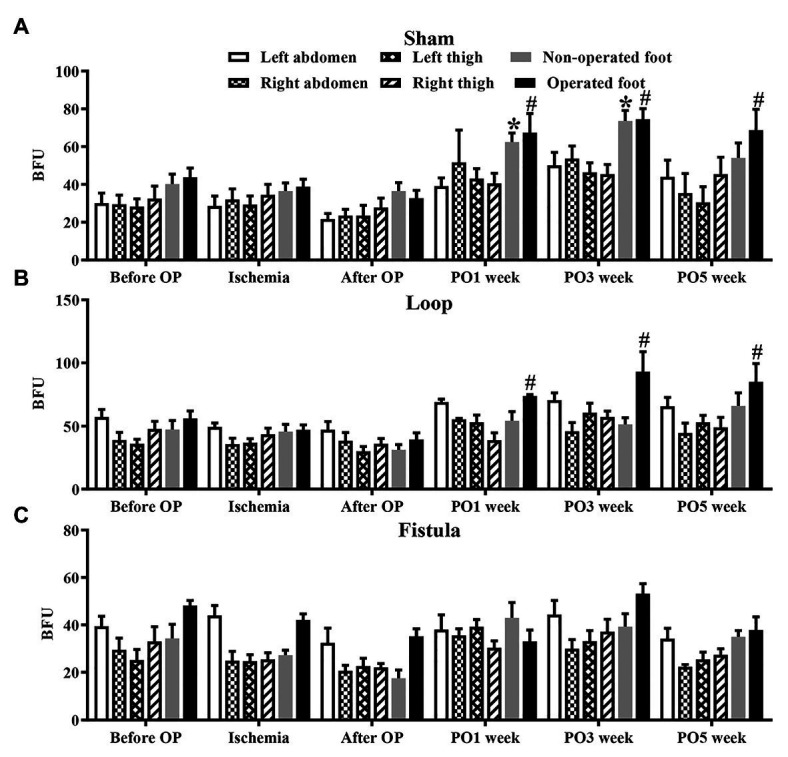
Results of microcirculatory measurements on various regions in the sham-operated **(A)**, loop **(B)**, and fistula **(C)** groups. *n* = 10, mean ± SEM, *p < 0.05 vs. non-operated foot after operation, #*p* < 0.05 vs. operated foot after operation.

**Figure 8:**
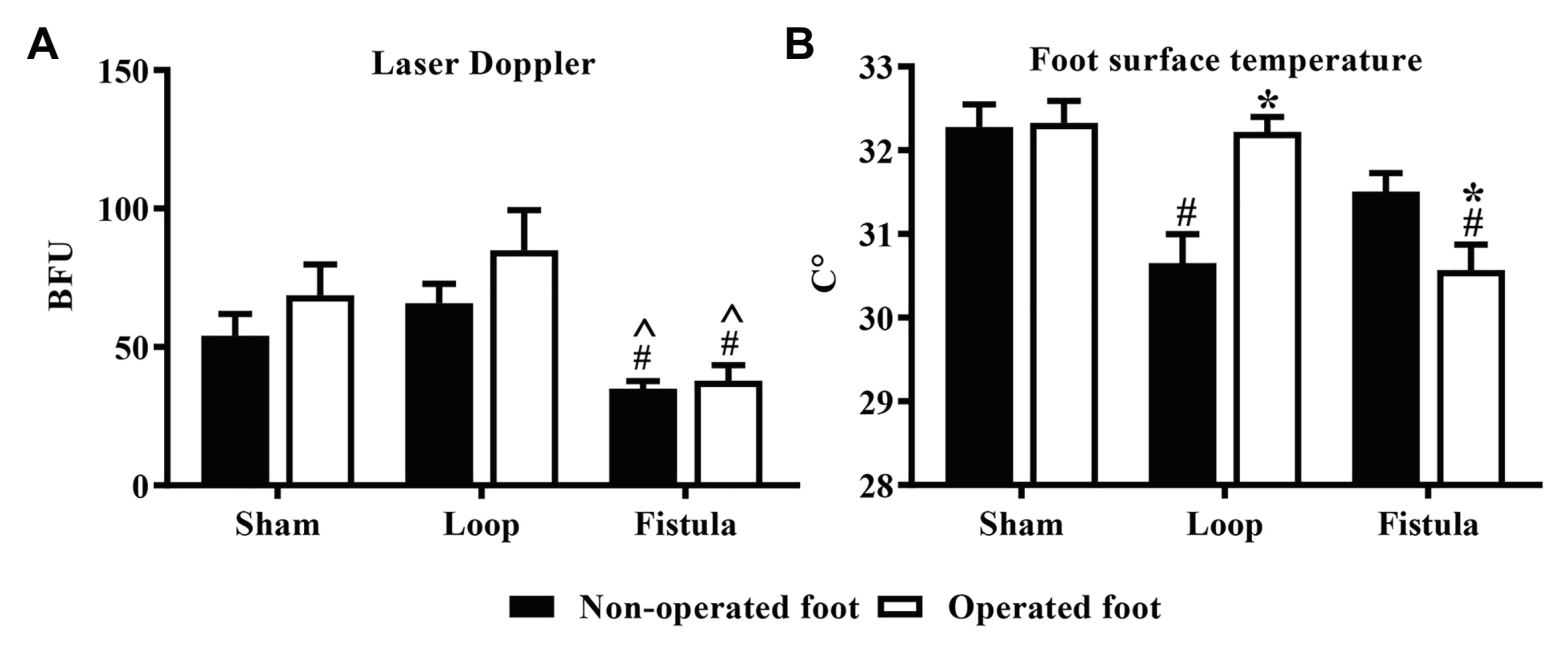
Additional microcirculatory **(A)** and foot (paw) skin surface measurements on the 5th postoperative week **(B)**. *n* = 10, mean ± SEM, ^*p* < 0.05 vs. loop group, #*p* < 0.05 vs. sham-operated group, *p < 0.05 vs. non-operated foot.

These results showed that, in the sham-operated group, as the rats grew, the microcirculatory values increased in both foot over the follow-up period. This increase was significant (Figure 7A). In the loop group, this kind of increase was missing from the non-operated foot (Figure 7B), and the fistula notably disturbed the microcirculation, because both foot microcirculations remained in lower levels (Figure 7C). Both hind limbs in the fistula group had significantly lower values than of the other two groups (Figure 8A). These results were supported by the foot surface temperature results as well. In the loop group, the non-operated foot surface temperature was significantly lower than the other foot, and it was also lower than the sham non-operated foot surface temperature (operated vs. non-operated: 32.22 ± 0.7 vs. 30.65 ± 1.35°C, *p* = 0.0009; sham vs. loop; 32.28 ± 1.15 vs. 30.65 ± 1.35°C, *p* = 0.0003; Figure 8B). Because of the steal mechanism caused by the fistula, we suspected that the surface temperature of the operated foot would be notably lower. The temperature measurement confirmed that the values were lower than on the other foot and the sham operated foot as well (operated vs. non-operated: 30.57 ± 1.4 vs. 31.51 ± 1.03°C, *p* = 0.0283; sham vs. fistula: 32.33 ± 1.11 vs. 30.57 ± 1.4°C, *p* < 0.0001; Figure 8B).

## Discussion

In this study, we examined the different hematological, hemorheological, and microcirculatory changes that may occur in the presence of an AVF and a loop-shaped venous graft. We found that the bigger surgical stress in case of the loop, caused the most of the early changes, but all of these normalized by the end of the follow-up period. The fistula surgery was not as stressful for the animals, therefore, lesser changes occurred at the beginning of the postoperative period. In contrast, the constant presence of the fistula caused several late alterations in the hematological and hemorheological parameters.

During the fistula maturation process, the endothelial tissue was stimulated by the pressure and shear stress (Davies, 2009). Response to that several molecular mediators can be secreted by the endothelial cell, which has great effect on the outcome of the histological changes that occur on the vessel. The exposure of vascular endothelium to shear forces in the normal value range stimulates endothelial cells to release agents with direct or indirect antithrombotic properties, such as prostacyclin, nitric oxide, thrombomodulin, etc., and can induce regeneration (Frangos et al., 1985; Rubanyi et al., 1986; Malek et al., 1994). However, if the shear forces are too low, thromboxane can be secreted alongside with other pro-thrombotic and vasoconstrictive molecules (Asif et al., 2006). Regarding the nature of the pressure and shear stress values, the vessel can react differently because inward or outward remodeling can occur (Browne et al., 2015). Inward remodeling is unfavorable because it can lead to the obstruction the fistula. This is modulated by pressure and shear stress (Gusic et al., 2005; Jia et al., 2015). High venous pressures are sensed by endothelial cells. In response, growth factors such as vascular endothelial growth factor (VEGF) are released, which can stimulate vascular regeneration but also vascular smooth muscle cell proliferation (Tisato et al., 2012; Lu et al., 2020). So it seems that high pressure and high shear stress is protective of the fistula and the opposite is promoting the failure the graft.

We suspected mechanical trauma to the RBCs because of two reasons. Reason one was that, after the surgery, the flow velocity of the blood increases significantly in a fistula, which highly elevates the shear forces inside a vessel (Anderson et al., 1977; Coppola and Caro, 2009). Even though the fistula matures and the diameter changed, the flow pattern in the fistula still remains pathological, because the blood flow is elevated thus the shear stress (Anderson et al., 1977). However, the arterialization process of the vein graft allows the vessel to adapt to the arterial conditions (Muto et al., 2010). The smaller diameter of the arterialized vein and the slower blood flow suggest that the shear stress is also reduced compared to the fistula. The second reason was that we anticipated intima hyperplasia, which could cause inner diameter and geometry changes (Subbotin, 2007; Browne et al., 2015). Stenosis changes the blood flow pattern from laminar toward turbulent flow, and this also elevates the mechanical stress on the RBCs (Clark, 1980; Liu, 2007; Vahidkhah et al., 2016). Steal mechanism was also expected, which could cause hypoperfusion on the operated limb (Mascia et al., 2010). Due to the vascular interventions, short‐ or long-term hypoperfusion or chronic ischemia could be developed, elevating the hypoxia-inducible factor 1 level, which in turn increases the erythropoietin production (Haase, 2013; Farsijani et al., 2016). The elevated erythropoietin increases RBC production and thus can alter hematological or even hemorheological values as well. Besides RBC production erythropoietin has other properties as well, it can affect the vessels as well (Santhanam et al., 2010). Elevated erythropoietin can also elevate the risk for AV fistula stenosis (Wärme et al., 2019).

Due to the stresses caused by the intervention, several parameters changed, but all of them are expected after surgery. The creation of the loop needs more dissection, and two anastomoses are made, which causes bigger blood loss. Since every surgery causes some level of inflammation (acute phase reaction), these together can explain the significant PO1 week changes compared to the other groups, and it is coherent with former studies (McKenzie and Laudicina, 1998; Arias et al., 2009; Toth et al., 2014). The MCV values showed a significant peak at the PO1 week in the loop group. We suspect that it was caused also by the bigger blood loss, because it is known in the literature that after notable blood loss the cell volume can increase due to the increased proliferating bone marrow cells (Nagao and Hirokawa, 2017). The platelet values also notably elevated in the loop group at the PO1 week, which was due to the blood loss and postoperative inflammation (Table 1; Santhosh-Kumar et al., 1991; Griesshammer et al., 1999). The unexpected result was that the fistula group values elevated by the end of the surgery. This side effect of the fistula is not well-known. We suspect that the chronic ischemic state of the operated limb through ischemic metabolites can increase the RBC levels as a compensatory mechanism (Haase, 2010, 2013). This effect may be only noticeable, because the fistula was placed on the femoral artery, which is able to “steal” significant amount of blood from the leg, without interfering with the blood supply of the other organs such as the kidney or the liver, unlike a carotid-jugular AVF (Ghanem et al., 2019). The prolonged inflammatory state of the rat in the fistula group caused by the chronic limb ischemia can increase the WBC count throughout the follow-up period (Table 2). This can explain why the values did not decrease in the fistula group contrary to the other two groups.

Since the blood is mixing in case of a fistula, we suspected some changes in the blood gas parameters. Pulmonary hypertension is expected in a presence of a fistula (Abassi et al., 2006). Contrary to other articles, a *p*CO_2_ levels did not decrease due to the pulmonary hypertension (Hoeper et al., 2007; Olsson et al., 2015). This could mean that in rats, in case of a femoral AVF, the pulmonary circulation is able to compensate. Because of the *p*CO_2_ values elevated, the *p*O_2_ values significantly decreased. The blood oxygenation level can affect deformability; therefore, this could also contribute to the PO5 week hemorheological changes that occurred in the fistula group (Uyuklu et al., 2009). Because the *p*O_2_ values were the highest in the fistula group at the PO5 week, unfortunately due to the elevated standard deviation, the difference was not significant. Also, the blood gas changes can induce the elevated RBC value as well. Comparing the venous blood to the mixed blood from the inferior caval vein, we observed a significant increase on a *p*O_2_ and glucose concentration. Oxygen controls the pulmonary capillaries’ constriction, the higher venous oxygen levels can add to the already existing pulmonary hypertension and decrease the lung perfusion.

In the RBC deformability values, we observed a significant impairment by the PO1 week in the loop group, correlating with the hematological findings. It would mean that the postoperative inflammation and the mechanical trauma caused by the not yet matured graft was damaging to the RBCs. During the graft maturation, the shear stress and turbulent flow decreases, therefore, the deformability values could normalize, as the vessels completely matured (within 4–6 weeks; Browne et al., 2015; Li et al., 2018). The EI_max_ values also normalized in every group, except in the fistula group, where it elevated significantly. The physiological shear stress values in the human body changes depending on the location. The shear stress in arterioles can be up to more than 5 Pa, in large veins around 0.1–0.5 Pa, however, these values are much higher in a fistula (Whitmore, 1969; Ene-Iordache and Remuzzi, 2012). Unlike the loop shaped graft, the fistula diameter enlarges during the maturation. This should moderately decrease the shear stress, but after the end of the maturation, the flow is still altered compared to the normal vessels (Siddiqui et al., 2017). The moderately elevated shear stress can improve the deformability by deliberation of NO from the cells (Meram et al., 2013; Simmonds et al., 2014). This supports our finding and suggests that the flow was still pathological at the PO5 week. As it was mentioned before, the chronic ischemic state of the hind limb caused elevation in the RBC count. We hypothesized that the newly formed young RBCs population altered the deformability values. It is known that the young RBCs have better deformability, thus it could elevate the tested deformability parameters, such as the EI_max_ values (Muravyov et al., 2002; Huisjes et al., 2018). The aggregation behaved the same way as the EI_max_ values, only the fistula group showed elevation. Since the microcirculation can be already damaged by a chronic disease, these small changes could seriously affect the distal microcirculation of the limbs (Libertiny and Hands, 1999; Savage et al., 2000; Rask-Madsen and King, 2013). We also noticed a strong correlation between the aggregation and the deformability values during the course of the experiment. This correlation was only notable above 9.49 Pa. Since the shear forces in the human body rarely go above 10 Pa, it may seem inadequate to examine forces above that level, but in case of arterial stenosis, the shear-stress could rise as high as 100 Pa (Samet and Lelkes, 1999; Waite and Fine, 2007).

The previously mentioned changes could be seen in the LD values as well. In the fistula group, where most of the hemorheological changes occurred, the microcirculation was the most impaired, not only on the operated leg but also on the non-operated leg. We suspect this is caused by the hemorheological changes, and that is why both hind limbs’ LD values were significantly lower than in the other two groups, because the steal mechanism caused by the fistula could decrease only the operated legs microcirculation. The hemorheological findings also suggest that, in case of a fistula, the microcirculation is more likely to be impaired.

## Conclusion

The presence of the fistula caused several changes in the hematological, hemorheological, and microcirculatory parameters by the end of the postoperative period. Even though the creation of the loop appeared more stressful for the animal, after 5 weeks of observation all parameters returned back to the normal values. In this rat model, the loop-shaped graft was better to preserve micro-rheological and microcirculatory state, and thus, the method might be a suitable alternative for hemodialysis when the AVF is not feasible.

## Data Availability Statement

Datasets are available on request: The raw data supporting the conclusions of this article will be made available by the authors, without undue reservation.

## Ethics Statement

The animal study was reviewed and approved by the Ethical permission registration Nr.: 25/2016/UDCAW University of Debrecen Animal Welfare Committee, University of Debrecen 4032 Debrecen Egyetem Tér 1. Hungary.

## Author Contributions

BS, lead author, PhD student, carried out the surgeries, took the blood samples, part of the statistics, and wrote the manuscript. BT, laboratory assistant, PhD student, helped with the laser Doppler measurements and analyzed the laser Doppler data. AV, laboratory assistant, PhD student, helped to prepare the blood samples and did most of the laboratory measurements of the blood samples. BB, laboratory assistant, PhD student, digitalized the data and assisted during the laboratory measurements. SG, PhD student, helped with the surgeries during the animal care and also did with the final revisions of the article. ZR, medical student, student research fellow, did several graphs of the manuscript and also helped with the statistical analysis of several parameters. MA-S, medical student, student research fellow, digitalized and summarized the blood gas and blood metabolite data and also helped during the postoperative measurements. NN, head of the department, supplied the materials and instrument for the experiment, helped with the design of the study, and did the final proof reading of the manuscript. All authors contributed to the article and approved the submitted version.

### Conflict of Interest

The authors declare that the research was conducted in the absence of any commercial or financial relationships that could be construed as a potential conflict of interest.

## References

[ref1] Aarts P. A. Banga J. D. van Houwelingen H. C. Heethaar R. M. Sixma J. J. (1986). Increased red blood cell deformability due to isoxsuprine administration decreases platelet adherence in a perfusion chamber: a double-blind cross-over study in patients with intermittent claudication. Blood 67, 1474–1481., PMID: 3516258

[ref2] Abassi Z. Nakhoul F. Khankin E. Reisner S. A. Yigla M. (2006). Pulmonary hypertension in chronic dialysis patients with arteriovenous fistula: pathogenesis and therapeutic prospective. Curr. Opin. Nephrol. Hypertens. 15, 353–360. 10.1097/01.mnh.0000232874.27846.37, PMID: 16775448

[ref3] Anderson C. B. Etheredge E. E. Harter H. R. Codd J. E. Graff R. J. Newton W. T. (1977). Blood flow measurements in arteriovenous dialysis fistulas. Surgery 81, 459–461., PMID: 847655

[ref4] Arias J. -I. Aller M. -A. Arias J. (2009). Surgical inflammation: a pathophysiological rainbow. J. Transl. Med. 7:19. 10.1186/1479-5876-7-19, PMID: 19309494 PMC2667492

[ref5] Asif A. Roy-Chaudhury P. Beathard G. A. (2006). Early arteriovenous fistula failure: a logical proposal for when and how to intervene. Clin. J. Am. Soc. Nephrol. 1, 332–339. 10.2215/CJN.00850805, PMID: 17699225

[ref6] Baskurt O. K. Hardeman M. R. Uyuklu M. Ulker P. Cengiz M. Nemeth N.. (2009). Parameterization of red blood cell elongation index--shear stress curves obtained by ektacytometry. Scand. J. Clin. Lab. Invest. 69, 777–788. 10.3109/00365510903266069, PMID: 19929721

[ref7] Bateman R. M. Sharpe M. D. Singer M. Ellis C. G. (2017). The effect of sepsis on the erythrocyte. Int. J. Mol. Sci. 18:1932. 10.3390/ijms18091932, PMID: 28885563 PMC5618581

[ref8] Binns R. L. Ku D. N. Stewart M. T. Ansley J. P. Coyle K. A. (1989). Optimal graft diameter: effect of wall shear stress on vascular healing. J. Vasc. Surg. 10, 326–337. 10.1016/0741-5214(89)90449-7, PMID: 2778897

[ref9] Browne L. D. Bashar K. Griffin P. Kavanagh E. G. Walsh S. R. Walsh M. T. (2015). The role of shear stress in arteriovenous fistula maturation and failure: a systematic review. PLoS One 10:e0145795. 10.1371/journal.pone.0145795, PMID: 26716840 PMC4696682

[ref10] Chai S. C. Sulaiman W. A. W. Saad A. Z. M. Rasool A. H. Shokri A. A. (2018). Skin microcirculatory changes in relation to arteriovenous fistula maturation. Indian J. Nephrol. 28, 421–426. 10.4103/ijn.IJN_402_17, PMID: 30647495 PMC6309389

[ref11] Chien S. (1987). Red cell deformability and its relevance to blood flow. Annu. Rev. Physiol. 49, 177–192. 10.1146/annurev.ph.49.030187.001141, PMID: 3551796

[ref12] Clark C. (1980). The propagation of turbulence produced by a stenosis. J. Biomech. 13, 591–604. 10.1016/0021-9290(80)90059-7, PMID: 7400187

[ref13] Coppola G. Caro C. (2009). Arterial geometry, flow pattern, wall shear and mass transport: potential physiological significance. J. R. Soc. Interface 6, 519–528. 10.1098/rsif.2008.0417, PMID: 19033138 PMC2696143

[ref14] Davies P. F. (2009). Hemodynamic shear stress and the endothelium in cardiovascular pathophysiology. Nat. Clin. Pract. Cardiovasc. Med. 6, 16–26. 10.1038/ncpcardio1397, PMID: 19029993 PMC2851404

[ref15] Ene-Iordache B. Remuzzi A. (2012). Disturbed flow in radial-cephalic arteriovenous fistulae for haemodialysis: low and oscillating shear stress locates the sites of stenosis. Nephrol. Dial. Transplant. 27, 358–368. 10.1093/ndt/gfr342, PMID: 21771751

[ref16] Farsijani N. M. Liu Q. Kobayashi H. Davidoff O. Sha F. Fandrey J.. (2016). Renal epithelium regulates erythropoiesis via HIF-dependent suppression of erythropoietin. J. Clin. Invest. 126, 1425–1437. 10.1172/JCI74997, PMID: 26927670 PMC4811147

[ref17] Fitts M. K. Pike D. B. Anderson K. Shiu Y. -T. (2014). Hemodynamic shear stress and endothelial dysfunction in hemodialysis access. Open Urol. Nephrol. J. 7, 33–44. 10.2174/1874303X01407010033, PMID: 25309636 PMC4189833

[ref18] Frangos J. A. Eskin S. G. McIntire L. V. Ives C. L. (1985). Flow effects on prostacyclin production by cultured human endothelial cells. Science 227, 1477–1479. 10.1126/science.3883488, PMID: 3883488

[ref19] Ghanem S. Somogyi V. Tanczos B. Szabo B. Deak A. Nemeth N. (2019). Modulation of micro-rheological and hematological parameters in the presence of artificial carotid-jugular fistula in rats. Clin. Hemorheol. Microcirc. 71, 325–335. 10.3233/CH-180411, PMID: 29914014

[ref20] Gnasso A. Carallo C. Irace C. Spagnuolo V. De Novara G. Mattioli P. L.. (1996). Association between intima-media thickness and wall shear stress in common carotid arteries in healthy male subjects. Circulation 94, 3257–3262. 10.1161/01.cir.94.12.3257, PMID: 8989138

[ref21] Green C. J. Knight J. Precious S. Simpkin S. (1981). Ketamine alone and combined with diazepam or xylazine in laboratory animals: a 10 year experience. Lab. Anim. 15, 163–170. 10.1258/002367781780959107, PMID: 7278122

[ref22] Griesshammer M. Bangerter M. Sauer T. Wennauer R. Bergmann L. Heimpel H. (1999). Aetiology and clinical significance of thrombocytosis: analysis of 732 patients with an elevated platelet count. J. Intern. Med. 245, 295–300. 10.1046/j.1365-2796.1999.00452.x, PMID: 10205592

[ref23] Gusic R. J. Myung R. Petko M. Gaynor J. W. Gooch K. J. (2005). Shear stress and pressure modulate saphenous vein remodeling ex vivo. J. Biomech. 38, 1760–1769. 10.1016/j.jbiomech.2004.10.030, PMID: 16023463

[ref24] Haase V. H. (2010). Hypoxic regulation of erythropoiesis and iron metabolism. Am. J. Physiol. Renal Physiol. 299, F1–F13. 10.1152/ajprenal.00174.2010, PMID: 20444740 PMC2904169

[ref25] Haase V. H. (2013). Regulation of erythropoiesis by hypoxia-inducible factors. Blood Rev. 27, 41–53. 10.1016/j.blre.2012.12.003, PMID: 23291219 PMC3731139

[ref26] Hammes M. (2015). Hemodynamic and biologic determinates of arteriovenous fistula outcomes in renal failure patients. Biomed. Res. Int. 2015:171674. 10.1155/2015/171674, PMID: 26495286 PMC4606083

[ref27] Hardeman M. R. Goedhart P. T. Shin S. (2007). “Methods in hemorheology” in Handbook of hemorheology and hemodynamics. Vol. 69 eds. Baskurt O. K. Meiselman H. J. Hardeman M. R. Rampling M. W. (Amsterdam, Washington, DC: IOS Press), 66–242.

[ref28] Hoeper M. M. Pletz M. W. Golpon H. Welte T. (2007). Prognostic value of blood gas analyses in patients with idiopathic pulmonary arterial hypertension. Eur. Respir. J. 29, 944–950. 10.1183/09031936.00134506, PMID: 17301100

[ref29] Holland C. K. Brown J. M. Scoutt L. M. Taylor K. J. (1998). Lower extremity volumetric arterial blood flow in normal subjects. Ultrasound Med. Biol. 24, 1079–1086. 10.1016/s0301-5629(98)00103-3, PMID: 9833575

[ref30] Hosseini S. M. Feng J. J. (2012). How malaria parasites reduce the deformability of infected red blood cells. Biophys. J. 103, 1–10. 10.1016/j.bpj.2012.05.026, PMID: 22828326 PMC3388224

[ref31] Hughes G. M. Kikuchi Y. (1988). Effects of temperature on the deformability of red blood cells of rainbow trout and ray. J. Mar. Biolog. 68, 619–625. 10.1017/S0025315400028757

[ref32] Huisjes R. Bogdanova A. van Solinge W. W. Schiffelers R. M. Kaestner L. van Wijk R. (2018). Squeezing for life—properties of red blood cell deformability. Front. Physiol. 9:656. 10.3389/fphys.2018.00656, PMID: 29910743 PMC5992676

[ref33] Jia L. Wang L. Wei F. Yu H. Dong H. Wang B.. (2015). Effects of wall shear stress in venous neointimal hyperplasia of arteriovenous fistulae. Nephrology 20, 335–342. 10.1111/nep.12394, PMID: 25581663

[ref34] Jose M. S. Nathan D. N. Seth M. V. Tanya G. Chen Sergey S. S. (2014). The relationship between red blood cell deformability metrics and perfusion of an artificial microvascular network. Clin. Hemorheol. Microcirc. 57, 275–289. 10.3233/CH-131719, PMID: 23603326 PMC3766416

[ref35] Jung F. Mrowietz C. Hiebl B. Franke R. P. Pindur G. Sternitzky R. (2011). Influence of rheological parameters on the velocity of erythrocytes passing nailfold capillaries in humans. Clin. Hemorheol. Microcirc. 48, 129–139. 10.3233/CH-2011-1392, PMID: 21876241

[ref36] Kameneva M. V. Antaki J. F. Shin S. (2007). “Mechanical trauma to blood” in Handbook of hemorheology and hemodynamics. Vol. 69 eds. Baskurt O. K. Meiselman H. J. Hardeman M. R. Rampling M. W. (Amsterdam, Washington, DC: IOS Press), 27–206.

[ref37] Karanasiou G. S. Gatsios D. A. Lykissas M. G. Stefanou K. A. Rigas G. A. Lagaris I. E.. (2015). Modeling of blood flow through sutured micro-vascular anastomoses. Annu. Int. Conf. IEEE Eng. Med. Biol. Soc. 2015, 1877–1880. 10.1109/EMBC.2015.7318748, PMID: 26736648

[ref38] Karino T. Goldsmith H. L. Motomiya M. Mabuchi S. Sohara Y. (1987). Flow patterns in vessels of simple and complex geometries. Ann. N. Y. Acad. Sci. 516, 422–441. 10.1111/j.1749-6632.1987.tb33063.x, PMID: 3439740

[ref39] Kiesewetter H. Jung F. Körber N. Wolf S. Kiehl R. Frank M.. (1986). Microcirculation and hemorheology of children with type I diabetes. Klin. Wochenschr. 64, 962–968., PMID: 3784448

[ref40] Ku D. N. (1997). Blood flow in arteries. Annu. Rev. Fluid Mech. 29, 399–434. 10.1146/annurev.fluid.29.1.399

[ref41] Kuzman D. Znidarcic T. Gros M. Vrhovec S. Svetina S. Zeks B. (2000). Effect of pH on red blood cell deformability. Pflugers Arch. 440(Suppl. 5), R193–R194., PMID: 28008537 10.1007/s004240000061

[ref42] Lecklin T. Egginton S. Nash G. B. (1996). Effect of temperature on the resistance of individual red blood cells to flow through capillary-sized apertures. Pflugers Arch. 432, 753–759. 10.1007/s004240050195, PMID: 8772123

[ref43] Li H. Jen S. Ramayya T. Bowers H. G. Rotem E. (2018). Unanticipated late maturation of an arteriovenous fistula after creation of separate graft access. Quant. Imaging Med. Surg. 8, 444–446. 10.21037/qims.2018.01.03, PMID: 29928609 PMC5989091

[ref44] Libertiny G. Hands L. (1999). Lower limb deep venous flow in patients with peripheral vascular disease. J. Vasc. Surg. 29, 1065–1070. 10.1016/S0741-5214(99)70247-8, PMID: 10359940

[ref45] Liu B. (2007). The influences of stenosis on the downstream flow pattern in curved arteries. Med. Eng. Phys. 29, 868–876. 10.1016/j.medengphy.2006.09.009, PMID: 17081795

[ref46] Lu J. Zhao Y. Zhang X. Li L. (2020). The vascular endothelial growth factor signaling pathway regulates liver sinusoidal endothelial cells during liver regeneration after partial hepatectomy. Expert Rev. Gastroenterol. Hepatol. 10.1080/17474124.2020.1815532 [Epub ahead of print] 32902336

[ref47] Malek A. M. Jackman R. Rosenberg R. D. Izumo S. (1994). Endothelial expression of thrombomodulin is reversibly regulated by fluid shear stress. Circ. Res. 74, 852–860. 10.1161/01.res.74.5.852, PMID: 8156632

[ref48] Mascia S. Spiezia S. Assanti A. De Nicola L. Stanzione G. Bertino V.. (2010). Ischemic steal syndrome in a hemodialysis patient: the roles of Doppler ultrasonography and dynamic Doppler studies in diagnosis and treatment selection. J. Ultrasound 13, 104–106. 10.1016/j.jus.2010.09.003, PMID: 23396797 PMC3552993

[ref49] McKenzie S. B. Laudicina R. J. (1998). Hematologic changes associated with infection. Clin. Lab. Sci. 11, 239–251., PMID: 10182113

[ref50] Meram E. Yilmaz B. D. Bas C. Atac N. Yalcin O. Meiselman H. J.. (2013). Shear stress-induced improvement of red blood cell deformability. Biorheology 50, 165–176. 10.3233/BIR-130637, PMID: 23863281

[ref51] Muravyov A. V. Draygin S. V. Eremin N. N. Muravyov A. A. (2002). The microrheological behavior of young and old red blood cells in athletes. Clin. Hemorheol. Microcirc. 26, 183–188., PMID: 12082249

[ref52] Muravyov A. V. Tikhomirova I. A. Mikhaylov P. V. Akhapkina A. A. Ostroumov R. S. (2018). Interrelations of hemorheological parameters and microcirculation in subjects with an increased blood pressure. Hum. Physiol. 44, 541–548. 10.1134/S0362119718050109

[ref53] Muto A. Model L. Ziegler K. Eghbalieh S. D. D. Dardik A. (2010). Mechanisms of vein graft adaptation to the arterial circulation: insights into the neointimal algorithm and management strategies. Circ. J. 74, 1501–1512. 10.1253/circj.cj-10-0495, PMID: 20606326 PMC3662001

[ref54] Nagao T. Hirokawa M. (2017). Diagnosis and treatment of macrocytic anemias in adults. J. Gen. Fam. Med. 18, 200–204. 10.1002/jgf2.31, PMID: 29264027 PMC5689413

[ref55] Nemeth N. Berhes M. Kiss F. Hajdu E. Deak A. Molnar A.. (2015). Early hemorheological changes in a porcine model of intravenously given *E. coli* induced fulminant sepsis. Clin. Hemorheol. Microcirc. 61, 479–496. 10.3233/CH-141914, PMID: 25536919

[ref56] Nemeth N. Deak A. Szentkereszty Z. Peto K. (2018). Effects and influencing factors on hemorheological variables taken into consideration in surgical pathophysiology research. Clin. Hemorheol. Microcirc. 69, 133–140. 10.3233/CH-189105, PMID: 29630533

[ref57] Nemeth N. Szabo A. (2017). “Microcirculation” in Advances in experimental surgery. eds. Chen H. Martins P. (Newyork, USA: Nova Science Publisher), 317–357.

[ref58] Oliver M. J. (2018). The science of fistula maturation. J. Am. Soc. Nephrol. 29, 2607–2609. 10.1681/ASN.2018090922, PMID: 30305311 PMC6218871

[ref59] Olsson K. M. Sommer L. Fuge J. Welte T. Hoeper M. M. (2015). Capillary pCO_2_ helps distinguishing idiopathic pulmonary arterial hypertension from pulmonary hypertension due to heart failure with preserved ejection fraction. Respir. Res. 16:34. 10.1186/s12931-015-0194-6, PMID: 25853979 PMC4358848

[ref60] Pandya A. N. Vaingankar N. Grant I. James N. K. (2003). End-to-side venous anastomoses….a patency test. Br. J. Plast. Surg. 56, 810–811. 10.1016/j.bjps.2003.08.007, PMID: 14615257

[ref61] Pike D. Shiu Y. -T. Somarathna M. Guo L. Isayeva T. Totenhagen J.. (2017). High resolution hemodynamic profiling of murine arteriovenous fistula using magnetic resonance imaging and computational fluid dynamics. Theor. Biol. Med. Model. 14:5. 10.1186/s12976-017-0053-x, PMID: 28320412 PMC5360029

[ref62] Rask-Madsen C. King G. L. (2013). Vascular complications of diabetes: mechanisms of injury and protective factors. Cell Metab. 17, 20–33. 10.1016/j.cmet.2012.11.012, PMID: 23312281 PMC3546345

[ref63] Rubanyi G. M. Romero J. C. Vanhoutte P. M. (1986). Flow-induced release of endothelium-derived relaxing factor. Am. J. Physiol. 250, H1145–H1149. 10.1152/ajpheart.1986.250.6.H1145, PMID: 3487253

[ref64] Samet M. M. Lelkes P. L. (1999). “The hemodynamic environment of the endothelium *in vivo* and its simulation *in vitro*” in Mechanical forces and the endothelium. ed. Lelkes P. L. (Amsterdsam: Harwood Academic Publishers), 1–32.

[ref65] Santhanam A. V. R. d’Uscio L. V. Katusic Z. S. (2010). Cardiovascular effects of erythropoietin: an update. Adv. Pharmacol. 60, 257–285. 10.1016/B978-0-12-385061-4.00009-X, PMID: 21081221 PMC3907121

[ref66] Santhosh-Kumar C. R. Yohannan M. D. Higgy K. E. al-Mashhadani S. A. (1991). Thrombocytosis in adults: analysis of 777 patients. J. Intern. Med. 229, 493–495. 10.1111/j.1365-2796.1991.tb00383.x, PMID: 2045755

[ref67] Savage C. O. S. Harper L. Cockwell P. Adu D. Howie A. J. (2000). Vasculitis. BMJ 320, 1325–1328. 10.1136/bmj.320.7245.1325 10807632 PMC1127317

[ref68] Siddiqui M. A. Ashraff S. Carline T. (2017). Maturation of arteriovenous fistula: analysis of key factors. Kidney Res. Clin. Pract. 36, 318–328. 10.23876/j.krcp.2017.36.4.318, PMID: 29285424 PMC5743041

[ref69] Simmonds M. J. Atac N. Baskurt O. K. Meiselman H. J. Yalcin O. (2014). Erythrocyte deformability responses to intermittent and continuous subhemolytic shear stress. Biorheology 51, 171–185. 10.3233/BIR-140665, PMID: 24948378

[ref70] Skoularigis J. Essop M. R. Skudicky D. Middlemost S. J. Sareli P. (1993). Frequency and severity of intravascular hemolysis after left-sided cardiac valve replacement with Medtronic Hall and St. Jude Medical prostheses, and influence of prosthetic type, position, size and number. Am. J. Cardiol. 71, 587–591. 10.1016/0002-9149(93)90516-f, PMID: 8438746

[ref71] Somogyi V. Peto K. Deak A. Tanczos B. Nemeth N. (2018). Effects of aging and gender on micro-rheology of blood in 3 to 18 months old male and female Wistar (Crl:WI) rats. Biorheology 54, 127–140. 10.3233/BIR-17148, PMID: 29562483

[ref72] Subbotin V. M. (2007). Analysis of arterial intimal hyperplasia: review and hypothesis. Theor. Biol. Med. Model. 4:41. 10.1186/1742-4682-4-41, PMID: 17974015 PMC2169223

[ref73] Telford R. D. Sly G. J. Hahn A. G. Cunningham R. B. Bryant C. Smith J. A. (2003). Footstrike is the major cause of hemolysis during running. J. Appl. Physiol. 94, 38–42. 10.1152/japplphysiol.00631.2001, PMID: 12391035

[ref74] Thiriet M. (2015). “Physiology and pathophysiology of venous flow” in PanVascular Medicine. ed. Lanzer P. (Berlin, Heidelberg: Springer), 569–589.

[ref75] Tisato V. Zauli G. Voltan R. Gianesini S. di Iasio M. G. Volpi I. (2012). Endothelial cells obtained from patients affected by chronic venous disease exhibit a pro-inflammatory phenotype. PLoS One 7:e39543. 10.1371/journal.pone.0039543 22737245 PMC3380919

[ref76] Toth C. Klarik Z. Kiss F. Toth E. Hargitai Z. Nemeth N.. (2014). Early postoperative changes in hematological, erythrocyte aggregation and blood coagulation parameters after unilateral implantation of polytetrafluoroethylene vascular graft in the femoral artery of beagle dogs. Acta Cir. Bras. 29, 320–327. 10.1590/S0102-86502014000500006, PMID: 24863320

[ref77] Uyuklu M. Meiselman H. J. Baskurt O. K. (2009). Effect of hemoglobin oxygenation level on red blood cell deformability and aggregation parameters. Clin. Hemorheol. Microcirc. 41, 179–188. 10.3233/CH-2009-1168, PMID: 19276515

[ref78] Vahidkhah K. Cordasco D. Abbasi M. Ge L. Tseng E. Bagchi P.. (2016). Flow-induced damage to blood cells in aortic valve stenosis. Ann. Biomed. Eng. 44, 2724–2736. 10.1007/s10439-016-1577-7, PMID: 27048168 PMC9924290

[ref79] Waite L. Fine L. (2007). Applied biofluid mechanics. 1st Edn. New York: McGraw-Hill Education.

[ref80] Wärme A. Hadimeri U. Hadimeri H. Nasic S. Stegmayr B. (2019). High doses of erythropoietin stimulating agents may be a risk factor for AV-fistula stenosis. Clin. Hemorheol. Microcirc. 71, 53–57. 10.3233/CH-180381, PMID: 29914013

[ref81] Whitmore R. L. (1969). Rheology of the circulation. New York: Pergamon Press, 1968.

[ref82] Yang X. -M. Liu J. Ji J. Xie J. (2014). Effects of dexmedetomidine on the deformability of erythrocytes in vitro and in anesthesia. Exp. Ther. Med. 7, 1631–1634. 10.3892/etm.2014.1633, PMID: 24926356 PMC4043598

[ref83] Yao C. Huang Y. Li X. Ruan P. (2003). Effects of pH on structure and function of single living erythrocyte. Chin. Sci. Bull. 48, 1342–1346. 10.1007/BF03184176

[ref84] Yen J. -H. Chen S. -F. Chern M. -K. Lu P. -C. (2014). The effect of turbulent viscous shear stress on red blood cell hemolysis. J. Artif. Organs 17, 178–185. 10.1007/s10047-014-0755-3, PMID: 24619800

[ref85] Zarins C. K. Zatina M. A. Giddens D. P. Ku D. N. Glagov S. (1987). Shear stress regulation of artery lumen diameter in experimental atherogenesis. J. Vasc. Surg. 5, 413–420., PMID: 3509594

